# Early‐stage predictors of the acute phase duration in uncomplicated COVID‐19 pneumonia

**DOI:** 10.1002/jmv.26281

**Published:** 2020-07-21

**Authors:** Claudio Carallo, Fabiola Pugliese, Cesare Tripolino, Lorenza Lenzi, Chiara Oliveri, Giada Fasani, Giovanni M. Guarrera, Walter Spagnolli, Susanna Cozzio

**Affiliations:** ^1^ Department of Chemical Engineering Imperial College London, South Kensington Campus London UK; ^2^ Department of Clinical and Experimental Medicine, Metabolic Diseases Unit Magna Græcia University Catanzaro Italy; ^3^ High‐Intensity Internal Medicine Unit Santa Maria del Carmine Hospital Rovereto Trento Italy; ^4^ Research in Advanced Neuro Rehabilitation, Istituto Sant'Anna Crotone Italy; ^5^ Internal Medicine plus Infectious Disease Units Santa Chiara Hospital Trento Italy; ^6^ APSS PA Trento Trento Italia

**Keywords:** COVID‐19 pneumonia, disease duration, hospital resources, hospital stay, in‐hospital, SARS‐CoV‐2

## Abstract

**Objective:**

In this study, we aimed to highlight the common early‐stage clinical and laboratory variables independently related to the acute phase duration in patients with uncomplicated coronavirus disease (COVID‐19) pneumonia.

**Methods:**

In hospitalized patients, the acute phase disease duration was followed using the Brescia‐COVID respiratory severity scale. Noninvasive ventilation was administered based on clinical judgment. Patients requiring oropharyngeal intubation were excluded from the study. For parameters to be measured at the hospital entrance, age, clinical history, National Early Warning Score 2 (a multiparametric score system), partial pressure of oxygen in arterial blood/fraction of inspired oxygen (P/F ratio), C‐reactive protein, and blood cell count were selected.

**Results:**

In 64 patients, age (direct relationship), P/F, and platelet number (inverse relationship) independently accounted for 43% of the acute phase duration of the disease (*P* < .001).

**Conclusions:**

For the first time, the present results revealed that the acute phase duration of noncomplicated pneumonia, resulting from severe acute respiratory syndrome coronavirus 2, is independently predicted from a patient's age, as well as based on the hospital entrance values of P/F ratio and peripheral blood platelet count.

## Highlights

- COVID‐19 has rapidly spread worldwide, straining the national health systems of several countries.
- It is needed to identify simple predictors of hospitalization duration to properly allocate medical resources.
- Age, P/F ratio, and platelet values predict acute phase duration of non‐complicated CoViD pneumonia.

## 1 BACKGROUND

Following the severe acute respiratory syndrome (SARS) owing to the coronavirus 2 (SARS‐CoV‐2) pandemic, a huge proportion of the general population is expected to require complex medical care as approximately 14% of patients develop the severe disease, requiring hospitalization.^1^

Therefore, to appropriately allocate medical resources, simple clinical or laboratory predictors identifying disease gravity at the first contact of patients with hospital facilities are required.

Apart from common disease signs and symptoms, organized in multiparametric score systems, optimal laboratory variables should be considered indices for systemic inflammation, including C‐reactive protein (CRP), white blood cell (WBC) count, and platelet count, known to be related to the hematologic impact of the disease, with PaO_2_ accounting for lung function. All these parameters were altered in coronavirus disease (COVID‐19) pneumonia. Furthermore, D‐dimer and procalcitonin could be allocated to later stage thrombotic and septic complications, respectively.^2^

Moreover, owing to the short time of disease outbreak, prediction models for COVID‐19 are poorly reported and present a high risk of bias, necessitating urgent collaborative efforts to develop more rigorous models.^3^

Predicting disease duration, particularly the acute phase, is crucial for superior clinical resource allocation. To date, a single, nonpeer‐reviewed chest computed tomography‐based model, has been reported as a predictor of short‐ and long‐term hospital stay (more than 10 days) in patients with pneumonia associated with SARS‐CoV‐2.^4^

Therefore, it is important to comprehensively identify predictors of disease duration, which could allow clinicians to perform appropriate patient risk stratification and resource allocation.^3^

In the present study, we aimed to identify early‐stage, commonly available clinical and laboratory variables related to the duration of the acute phase in uncomplicated COVID‐19 pneumonia.

## 2 METHODS

### 2.1 Study design

This study was performed using clinical data collected from patients with COVID‐19 pneumonia, discharged to go home by the Internal Medicine plus Infectious Disease Units of Santa Chiara Hospital in Trento (Trento, Italy), and by the High‐Intensity Internal Medicine Unit of Santa Maria del Carmine Hospital in Rovereto (Trento, Italy), between 1 March and 18 April 2020. Data for the parameters used for predictive analyses were obtained on the first day of hospital admission. Given the exclusion of patients with complications during in‐hospital stay, and the rigorous protocol for admission and discharge, the length of stay was assimilated to the acute phase duration of this disease.

### 2.2 Ethics

In the present investigation, data were collected and examined anonymously (nonsensitive data) and extracted as aggregated from the electronic health record. Patients had previously provided informed consent for treatment (https://www.apss.tn.it/documents/10180//179124//Informativa+fascicolo+sanitario+elettronico+FSE.pdf). The identity of all subjects was deleted, and random identification numbers were used to analyze the data.

### 2.3 Subjects

The inclusion criteria were as follows: evidence of interstitial pneumonia by at least a standard chest X‐ray; diagnosis of COVID‐19 infection with RNA detection of SARS‐CoV‐2 from nasal and/or pharyngeal swab specimens; disease duration less than 1 month, hospital stay greater than 2 days; age greater than 18 years.

The exclusion criteria were oropharyngeal intubation before, during, or after admission; a history of active cancer in the last year; chronic kidney disease stage IV‐V; hepatic cirrhosis; heart failure New York Heart Association class greater than II; chronic obstructive pulmonary disease; recent surgical intervention or bone fractures; and possibly pregnant women.

### 2.4 Definition of variables

For all subjects, clinical, radiological, and laboratory examinations were performed during the first 24 hours of admission.

Body mass index (BMI) was computed as weight (in kg) divided by height (in m^2^). Obesity was defined as a BMI of greater than or equal to 30 kg/m^2^. Diabetes was defined as fasting blood glucose greater than or equal to 126 mg/dL on two occasions, and/or the use of antidiabetic agents. Hyperlipidemia was defined as total cholesterol and/or triglycerides greater than or equal to 200 mg/dL, and/or the use of lipid‐lowering drugs. Hypertension was defined as systolic blood pressure/diastolic blood pressure greater than or equal to 140/90 mm Hg, and/or the use of antihypertensive agents. Subjects who smoked regularly during the previous 12 months were classified as smokers.

Laboratory examinations included serum CRP, complete blood count, and partial pressure of oxygen in arterial blood (PaO_2_). The fraction of inspired oxygen (FiO_2_) was annotated. Then, the PaO2/FiO2 ratio (P/F ratio) was calculated.

Arterial blood saturation with oxygen (SpO_2_) was measured by pulse oximetry. The National Early Warning Score 2 (NEWS2), a multiparametric score system, was calculated from respiratory rate, oxygen saturation with ambient air conditions, temperature, blood pressure, pulse/heart rate, hypercapnic respiratory failure, room air or supplemental O_2_, level of consciousness, or new confusion.^5^

### 2.5 Therapies

Immediately after admission, all patients were treated with hydroxychloroquine 200 mg capsules bid, and antiviral agents such as lopinavir/ritonavir 400 + 100 mg cp bid or 200 + 50 mg cp bid if gastrointestinal side effects were present.

Noninvasive ventilation (NIV) was administered depending on the Brescia‐COVID respiratory severity scale (BCRSS) using continuous positive airway pressure.

Tocilizumab therapy was administered as 324 mg subcutaneously, repeated after 16 hours, based on clinical judgment (severe pneumonia and rapidly evolving respiratory insufficiency, in elderly patients or those at a higher risk of developing severe disease and acute respiratory distress syndrome).^6^

All patients underwent anticoagulant treatment, defined as receiving enoxaparin from 4000 to 8000 UI/d as low molecular weight heparin; the dose range was based on clinical judgment.

### 2.6 Admission and discharge

Patients with confirmed COVID‐19 pneumonia were evaluated before admission to BCRSS.^6^ Validated criteria were:

1. Patient wheezing OR unable to speak in full sentences while resting/with minimal effort;
2. Respiratory rate greater than 22 breaths/min;
3. PaO_2_ less than 65 mm Hg or SpO_2_ less than 90%;
4. Significantly worsening repeated chest rays.

If the patient presented no or one criterion, he returned home for active remote surveillance.

If the patient presented two criteria, he was admitted to internal medicine facilities and received medical treatment, becoming eligible for this study.

If the patient presented three or more criteria, he was admitted to the High‐Intensity Internal Medicine for additional NIV, becoming eligible for this study. If recovered, he was transferred for medical treatment to the internal medicine facilities; otherwise, he was moved to an intensive care unit for oropharyngeal intubation, exiting the present study protocol.

Hospital discharge was performed when the patient presented no BCRSS criteria (zero score), returning home for treatment through eventual on‐call remote facilities. RNA detection of SARS‐CoV‐2 from nasal and/or pharyngeal swab specimens was repeated the day before and at discharge. If one or both were positive, the patient was instructed for out‐of‐hospital isolation until two tests were negative over a 2‐day span.

### 2.7 Statistical analyses

Statistical analyses were performed using PASW 18.0 for Windows. The normality of distribution was assessed using the Shapiro‐Wilk test. All continuous variables presented normal distribution except for CRP, WBC count, lymphocytes, and neutrophil count, and platelet number. Non‐normally distributed variables were log‐transformed before analyses. Pearson or Spearman's correlation coefficients were used as appropriate, to test the correlation between continuous variables in simple and multiple approaches. Multiple regression analyses were performed to test the independent association between disease duration and clinical and biochemical variables. In detail, variables were entered into two blocks, one for preexistent clinical conditions and one for both clinical and biochemical disease presentations. In the first block, age, sex, obesity, diabetes mellitus, hypertension, hyperlipidemia, smoking, and previous cardiovascular disease were included; in the second block, the P/F ratio, NEWS2 index, CRP, platelet count, lymphocyte count, and neutrophil counts were included. Statistical significance was set at *P* < .05.

## 3 RESULTS

Overall, 64 patients were recruited for the present analysis. Table 1 presents the clinical and biochemical characteristics of the patients. The patients were mainly middle‐aged men, and the mean NEWS2 score was 4.8 ± 3. The main disease duration was 7.5 ± 4.2 days. The prevalence of cardiovascular risk factors was as follows: 46% for hypertension, 21% for hyperlipidemia, 24% for obesity, and 11% for diabetes. Smokers constituted 3% of the group.

**Table 1:** Clinical features of subjects included into the study

Variables	Results
Age, y	62 ± 11.6
Male sex, %	72
Hypertension, %	46
Dyslipidemia, %	21
Obesity, %	24
Smoking, %	3
Diabetes mellitus, %	11
NEWS index	4.8 ± 3
P/F ratio, mm Hg	264 ± 108
Disease duration, d	7.5 ± 4.2
White cells count, #/μL	7231 ± 3806
O_2_‐saturation, %	93 ± 4.2
Neutrophils, 10^3^/μL	4690 ± 3503
Lymphocytes, #/μL	1200 ± 470
Platelets, #/μL	221 796 ± 113 975
CRP, mg/L	79 ± 68

Abbreviations: CRP, C‐reactive protein; NEWS2, National Early Warning Score 2; P/F ratio, partial pressure of oxygen in arterial blood (PaO_2_)/fraction of inspired oxygen (FiO_2_).

Following simple regression analyses, disease duration was found to be significantly associated with age (*r* = .29, *P* = .018), NEWS2 score (*r* = .46, *P* < .001), P/F ratio (*r* = −.58, *P* = .018), CRP (*r* = .27, *P* = .035), and lymphocyte count (*r* = −.27, *P* = .030).

To further investigate the association between disease duration and clinical and biochemical variables, a multiple regression analysis was performed. Disease duration was used as the dependent variable, while independent variables were entered into two blocks: age, sex, and clinical history variables in the first block; hospital entrance clinical parameters in the second block. As displayed in Table 2, the variables that independently associated with disease duration were age (direct relationship), P/F, and platelet number (inverse relationship). This model alone, with all the parameters measured at the hospital entrance, explained more than 43% of the length of the acute phase of the disease (*P* < .001).

**Table 2:** Results of multiple linear regression analysis (*n* = 64)

	*β* coefficient	*R* ^2^	*t*	*P*
Entered variables				
Age	.11	0.11	2.47	.017
P/F ratio	−.59	0.37	−4.90	.001
Platelets	−.25	0.43	−2.22	.031
Excluded variables				
Sex	.06	…	0.54	.58
Diabetes	−.02	…	−0.23	.81
Hypertension	.03	…	0.02	.97
Obesity	−.01	…	−0.12	.90
Previous CVD	−.03	…	−0.29	.77
NEWS	.19	…	1.10	.27
CRP	.14	…	1.21	.23
Lymphocyte count	−.15	…	−1.29	.20
Neutrophil count	.09	…	0.60	.55

*Note:* Dependent variable: acute phase COVID‐19 pneumonia duration. The model presents an *R* ^2^ value of 0.433, with a *P* value of .001.

Abbreviations: CRP, C‐reactive protein; CVD, cardiovascular diseases; NEWS2, National Early Warning Score 2; P/F ratio, partial pressure of oxygen in arterial blood (PaO_2_)/fraction of inspired oxygen (FiO_2_).

## 4 DISCUSSION

Since its first appearance in China, COVID‐19 has rapidly spread worldwide, straining the national health systems of several countries. Hence, it is crucial to identify simple clinical or laboratory predictors of hospitalization duration at the first contact of patients with hospital facilities, to appropriately allocate medical resources. Our investigation demonstrated that patient age, P/F ratio at admission, and platelet count predicted the acute phase duration of noncomplicated SARS‐CoV‐2 pneumonia.

The mean disease duration was approximately 1 week. Other studies have utilized different admission and discharge protocols. However, data from a large cohort of 1420 patients from 18 European hospitals are available^7^; however, this data pertains to the duration of *symptoms* of mild‐to‐moderate COVID‐19 (962 females, mean age 39.17 ± 12.0 years, 30.7% of health care workers), with symptoms disappearing after 11.5 ± 5.7 days. This difference is attributed to patient being discharged after the acute phase of pneumonia, only when alarm symptoms and signs such as dyspnea and oxygen desaturation disappeared, while still presenting symptoms such as cough or asthenia.

Several other reports investigated the duration of *virus shedding*; in one such study, virus shedding was found to be significantly longer in men than in women and in older age groups, partly explaining the high rate of severe illness in men over 60 years of age.^8^ Furthermore, US hospitalization rates were higher (13.8/100 000 population) among adults aged greater than or equal to 65 years.^9^ In the present study, age was the only independent predictor of the acute phase disease duration among the general clinical status preceding the disease.

In terms of literature regarding *disease aggravation*, one study has highlighted risk factors such as male sex, older age, diabetes, cardiovascular diseases, chills, dyspnea, and SatO_2_ value of less than or equal to 93%, WBC counts greater than 10 × 10^9^/L, and large consolidated opacities on computed tomography images.^10^ In another report recruiting 189 patients, old age and higher serum lactate dehydrogenase, CRP, the coefficient of variation of red blood cell distribution width, blood urea nitrogen, direct bilirubin, and lower albumin have been associated with severe COVID‐19.^11^ In a meta‐analysis, a low platelet count has been associated with up to a fivefold enhanced risk for severe COVID‐19.^12^ In a novel scoring model, named as CALL, 208 patients were divided into the stable group (*n* = 168, 80.8%) and progressive group (*n* = 40,19.2%): comorbidity, older age, lower lymphocyte count, and higher lactate dehydrogenase at presentation were reported as independent high risk factors for COVID‐19 progression.^13^

The results of our investigation recorded age, P/F ratio, and platelet count as predictors of hospital stay. The coefficients of simple regression analyses were poor. We speculate that the low number of subjects and the presence of several confounding factors might account for these results. To the best of our knowledge, only a few studies have analyzed this issue.

Liu et al^14^ have observed that lymphopenia was associated with prolonged hospitalization in COVID‐19 patients, whereas serum CRP and lymphocyte count were associated with adverse outcomes. In a recent study, Moriconi et al^15^ have demonstrated that BMI, CRP, and lymphocyte count were associated with longer hospital stays in obese patients.

Our results appear consistent with those of Qu et al.^16^ In their study, the authors observed that platelet and platelet to lymphocyte ratios are associated with a longer hospital stay in COVID‐19 patients. During inflammatory conditions, such as COVID‐19, cytokines (particularly interleukin‐6) stimulate the production of thrombopoietin and, in turn, the generation of megakaryocytes (and then platelets). In this context, platelets could be considered as indicators of systemic inflammation, correlating with a longer hospital stay. Additionally, the association between platelet count and poor outcomes was investigated in other studies. In 1476 consecutive COVID‐19 patients, the reported mortality was 92.1%, 61.2%, 17.5%, and 4.7% for the (0, 50), (50, 100), (100‐150), and (150‐) platelet groups; the first group presented a 13.68 relative risk when compared with the last one.^17^

In our investigation, age was associated with prolonged hospitalization. Typically, older individuals present a diminished immune response, reduced mucociliary clearance, and have multiple comorbidities than younger individuals. Moreover, several studies have considered age; in a multivariate logistic analysis, age (odds ratio = 1.06) and CD4 T cell count (odds ratio = 0.55/100 cells/μL increase) were independently associated with oropharyngeal intubation.^18^ Fatality during COVID‐19 shows a strong age gradient in the risk of death, up to a maximum of 18.4% in those aged 80 years or older.^19^ Among 1408 Chinese patients,^20^ if age was greater than or equal to 80, the mortality rate was 14.8%, more than that observed in patients with hypertension (6.0%) and diabetes (7.3%). Reportedly, older age, higher Sequential Organ Failure Assessment score, and elevated D‐dimer levels at admission were risk factors for death in adult patients with COVID‐19.^21^

Finally, in our research, the P/F ratio on admission demonstrated a stronger relationship with hospitalization when compared with other variables.

Moreover, P/F was considered in mortality studies; its mean value was reportedly 290 mm Hg in 67 patients who died during COVID‐19 pneumonia and 400mm Hg in 1032 survivors.^22^

## 5 CONCLUSIONS

The present data, for the first time, demonstrated that age, and easily available measurements of P/F ratio and peripheral blood platelets, can predict almost half the variabilities observed in the acute disease phase duration of uncomplicated pneumonia induced by SARS‐CoV‐2. These variables might be considered an easy‐to‐obtain information package that could guide medical resource allocation, presenting novel candidates for new prediction models.

## CONFLICT OF INTERESTS

The authors declare that there are no conflict of interests.

## AUTHOR CONTRIBUTIONS

Study concept and design: CC, FP, and CT. Study collection: FP, LL, and CO. Acquisition and analysis of data: FP, GF, and GMG. Drafting and writing of the manuscript: CC and CT. Revision of the manuscript: WS and SC.
